# Inactivation of Fructose-1,6-Bisphosphate Aldolase Prevents Optimal Co-catabolism of Glycolytic and Gluconeogenic Carbon Substrates in *Mycobacterium tuberculosis*

**DOI:** 10.1371/journal.ppat.1004144

**Published:** 2014-05-22

**Authors:** Susan Puckett, Carolina Trujillo, Hyungjin Eoh, Joeli Marrero, John Spencer, Mary Jackson, Dirk Schnappinger, Kyu Rhee, Sabine Ehrt

**Affiliations:** 1 Department of Microbiology and Immunology, Weill Cornell Medical College, New York, New York, United States of America; 2 Department of Medicine, Weill Cornell Medical College, New York, New York, United States of America; 3 Mycobacteria Research Laboratories, Department of Microbiology, Immunology and Pathology, Colorado State University, Fort Collins, Colorado, United States of America; National Institutes of Health, United States of America

## Abstract

Metabolic pathways used by *Mycobacterium tuberculosis* (*Mtb*) to establish and maintain infections are important for our understanding of pathogenesis and the development of new chemotherapies. To investigate the role of fructose-1,6-bisphosphate aldolase (FBA), we engineered an *Mtb* strain in which FBA levels were regulated by anhydrotetracycline. Depletion of FBA resulted in clearance of *Mtb* in both the acute and chronic phases of infection *in vivo*, and loss of viability *in vitro* when cultured on single carbon sources. Consistent with prior reports of *Mtb's* ability to co-catabolize multiple carbon sources, this *in vitro* essentiality could be overcome when cultured on mixtures of glycolytic and gluconeogenic carbon sources, enabling generation of an *fba* knockout (Δ*fba*). *In vitro* studies of Δ*fba* however revealed that lack of FBA could only be compensated for by a specific balance of glucose and butyrate in which growth and metabolism of butyrate were determined by *Mtb's* ability to co-catabolize glucose. These data thus not only evaluate FBA as a potential drug target in both replicating and persistent *Mtb*, but also expand our understanding of the multiplicity of *in vitro* conditions that define the essentiality of *Mtb's* FBA *in vivo*.

## Author Summary

The development of new chemotherapies targeting *Mycobacterium tuberculosis* (*Mtb*) will benefit from genetic evaluation of potential drug targets and a better understanding of the pathways required by *Mtb* to establish and maintain chronic infections. We employed a genetic approach to investigate the essentiality of fructose-1,6-bisphosphate aldolase (FBA) for growth and survival of *Mtb in vitro* and in mice. A conditional *fba* mutant revealed that *Mtb* requires FBA for growth in the acute phase and for survival in the chronic phase of mouse infections. *In vitro* essentiality of *fba* was strictly condition-dependent. An FBA deletion mutant (Δ*fba*) required a balanced combination of carbon substrates entering metabolism above and below the FBA-catalyzed reaction for growth and died in media with single carbon sources and in mouse lungs. Death of Δ*fba in vitro* was associated with the perturbation of intracellular metabolites. These studies highlight how a conditional *fba* mutant helped identify conditions in which FBA is dispensable for growth of *Mtb*, evaluate FBA as a potential target for eliminating persistent bacilli and offer insight into metabolic regulation of carbon co-catabolism in *Mtb*.

## Introduction

Metabolism is an important aspect of all host–pathogen interactions [1]. *Mycobacterium tuberculosis* (*Mtb*) has evolved to replicate and survive for decades within diverse host granulomas, including caseating, fibrotic and cavitating lesions, each providing a different environment [2], [3]. *Mtb*'s ability to adapt to multiple environments may in part be attributed to its metabolic flexibility and its capacity to efficiently catabolize multiple carbon sources simultaneously [4]–[6]. Fatty acids and lipids are major carbon sources available to *Mtb in vivo* [7]–[10], but *Mtb* also has access to glucose and glycolytic 3-carbon compounds, requires trehalose import for virulence and can utilize CO_2_ as source of carbon [6], [11]–[13]. *Mtb* lacks classical carbon catabolite repression and it remains to be identified how co-catabolism of multiple carbon sources is regulated to achieve optimal growth [4].

Knowledge about *Mtb's* metabolism benefits the understanding of tuberculosis pathogenesis and can identify potential new targets for chemotherapeutic interventions, as *Mtb* mutants lacking metabolic enzymes are among the most attenuated in the mouse model of tuberculosis [8], [10], [14]–[17]. Fructose bisphosphate aldolase (FBA) is central to glycolysis and gluconeogenesis (Figure S1) and has been the focus of structural, enzymatic, and drug developmental studies [18]–[22]. Evidence supporting FBA's requirement for optimal growth came from the failure to isolate mutants with transposon insertions in *fba*, the gene encoding FBA, in standard *in vitro* culture conditions [23]. Also, previous attempts to delete *fba* from the *Mtb* chromosome by homologous recombination have failed [18], [19]. A conditional *fba* mutant generated in the attenuated H37Ra strain revealed that growth in either glucose- or succinate-containing media was strictly dependent upon induction of FBA expression, demonstrating that FBA is an essential enzyme for both glycolysis and gluconeogenesis [19]. Moreover, FBA is expressed in *Mtb* during mouse and guinea pig infections, and increased amounts were identified in culture filtrates during hypoxia, a condition which persistent *Mtb* is thought to encounter in the host [19], [24]. FBA has recently been shown to be secreted by *Mtb* and to bind host plasminogen, potentially enabling FBA to play a metabolism-independent role in host-pathogen interactions [19]. Importantly, *Mtb* FBA differs from human FBA by its reaction mechanism. FBAs catalyze the reversible cleavage of fructose-1,6-bisphosphate (FBP) to yield dihydroxyacetone phosphate (DHAP) and glyceraldehyde 3-phosphate (G3P). However, class I FBAs such as the human enzyme produce a Schiff-base reaction intermediate whereas class II FBAs, to which the *Mtb* enzyme belongs, require a divalent metal cation such as Zn^2+^ to stabilize the enolate intermediate [25]. Although some bacteria express both class I and class II enzymes, *Mtb* lacks an annotated class I FBA [19], [26]. Class I FBA activity in *Mtb* has been reported in earlier studies, but could not be detected by others [19], [27], [28]. The difference in catalytic mechanism of FBA from humans compared to that of *Mtb* enabled the design of bacteria-targeting class II-specific FBA inhibitors and work is ongoing to improve their efficacy [20], [22], [29], [30].

Our goal was to investigate the importance of *Mtb's* FBA in acute and chronic mouse infections and to further characterize the basis for its *in vitro* essentiality. To achieve these goals we generated *Mtb* strains in which FBA expression was tightly regulated by a tunable dual-control (DUC) genetic switch that combines transcriptional silencing and controlled protein depletion [31]. We found that essentiality of FBA was condition-dependent, and could be overcome by availability of two carbon sources entering metabolism above and below the FBA-catalyzed step. However this was dependent on the specific ratio of the glycolytic and gluconeogenic carbon sources, because metabolism of butyrate by a strain lacking *fba* was dependent on *Mtb's* ability to efficiently co-catabolize glucose. *Mtb* relied on FBA for both growth during acute and persistence during chronic mouse infections.

## Results

### FBA is required for growth in glycolytic and gluconeogenic carbon sources and for growth and persistence in mice

**Figure 1:**
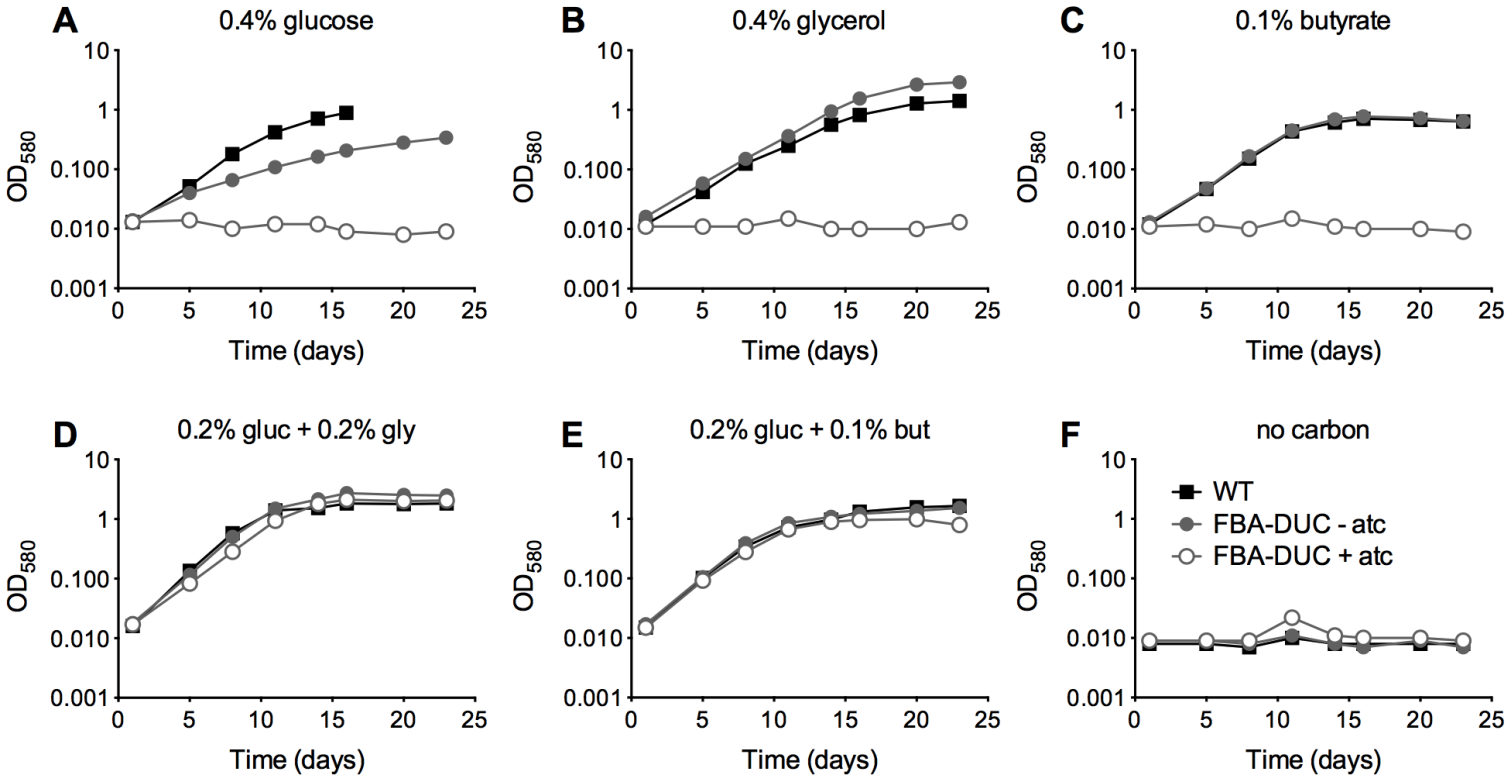
FBA depletion inhibits growth of *Mtb* in single carbon sources, but not in the presence of both a glycolytic and a gluconeogenic carbon source. Growth of WT (black squares) and FBA-DUC in the absence (grey circles) and presence (open circles) of 500 ng/ml atc in carbon defined media containing (**A**) 0.4% glucose (**B**) 0.4% glycerol (**C**) 0.1% butyrate (**D**) 0.2% glucose and 0.2% glycerol (**E**) 0.2% glucose and 0.1% butyrate and (**F**) no carbon. Bacteria were cultured in 25 cm^2^ flasks. Data are representative of at least two independent experiments.

To investigate the role of FBA *in vitro* and *in vivo* we generated an *Mtb* strain in which expression of FBA is regulated by the recently described DUC switch [31], so that anhydrotetracycline (atc) or doxycycline (doxy) trigger transcriptional repression of the *fba* gene and simultaneous degradation of the FBA protein. We first introduced a second copy of *fba* with a strong promoter (P_smyc_-*fba*) [32] via an integrative plasmid into the *Mtb* chromosome and then deleted the native copy. After confirming *fba* deletion by Southern blot (Figure S2), we generated FBA-DUC by replacing P_smyc_-*fba* with a DAS+4-tagged *fba* gene, whose transcription was controlled by a TetR-regulated promoter. The DAS+4-tag allowed for proteolytic inactivation of FBA. Immunoblot analysis confirmed FBA depletion upon addition of atc (Figure S3). Similar to previous findings with an H37Ra FBA-TetON mutant [19], growth of H37Rv FBA-DUC with single carbon sources was inhibited when FBA was depleted by the addition of atc (Figure 1A–C). Unexpectedly, however, growth of FBA-DUC was unaffected by atc in media containing glucose and a second carbon source, such as butyrate, or glycerol (Figure 1D,E).

**Figure 2:**
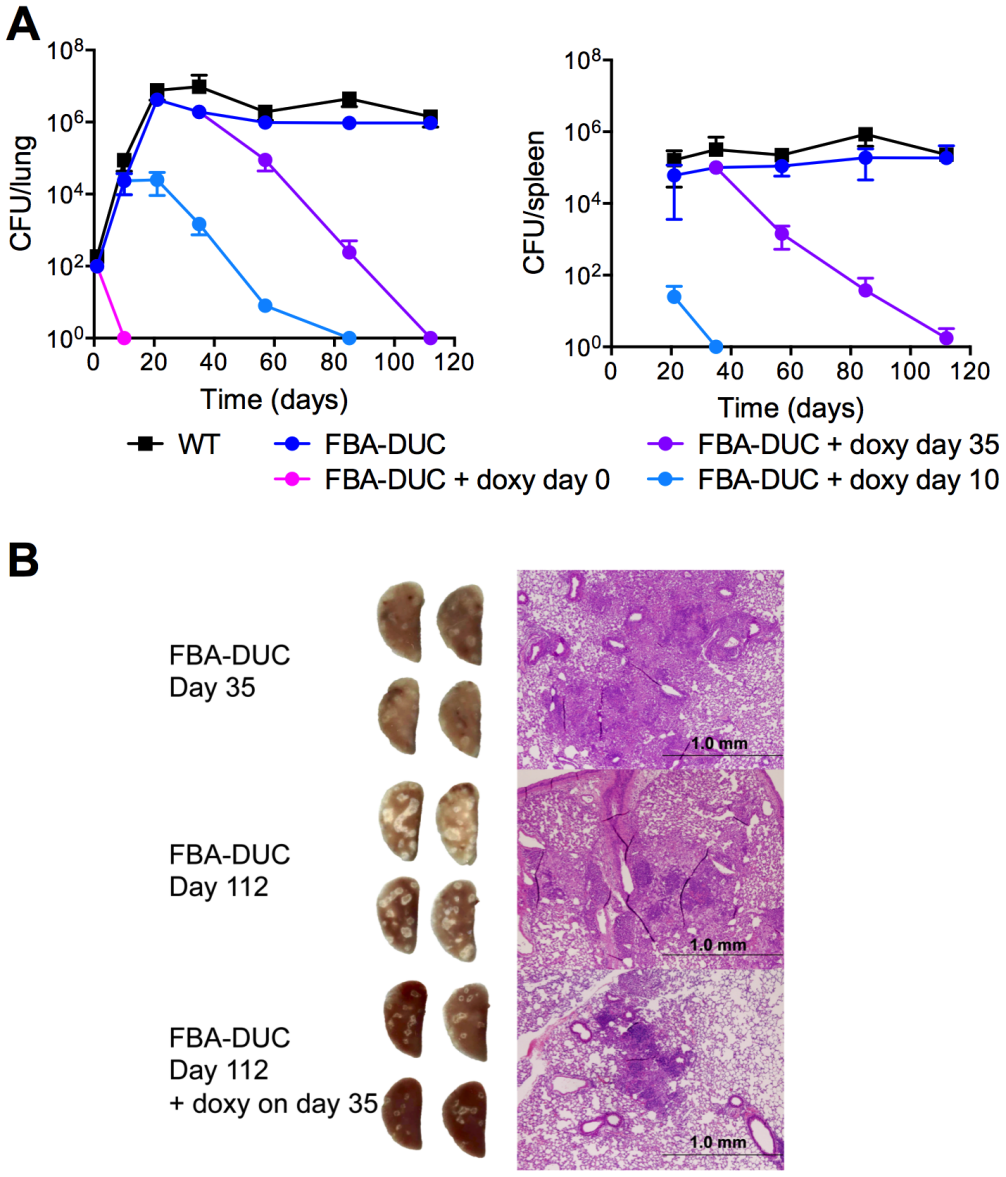
FBA is required for replication and persistence of *Mtb* in mice. (**A**) Growth and survival of WT (squares) and FBA-DUC (circles) in mouse lungs (left panel) and spleens (right panel). Mice infected with FBA-DUC received doxy-containing food from the day of infection (day 0), day 10, day 35 or not at all as indicated. CFU were not detected in spleens from mice infected with FBA-DUC and treated with doxy starting day 0. The limit of detection was 4 CFU in lungs and spleens. Data are means ± SD of four mice, except for three data points which derive from 3 or 2 mice due to the appearance of atc/doxy resistant revertants in the lungs (day 57 FBA-DUC+doxy day 10, day 85 FBA-DUC+doxy day 10 and day 112 FBA-DUC+doxy day 35). (**B**) Gross pathology and H&E staining of lung sections from mice infected with FBA-DUC. Lungs were isolated at day 35 (upper panel) and day 112 (middle and lower panel) from mice not treated and administered doxy starting day 35 post infection. A second short course infection experiment reproduced the phenotype of FBA-DUC in mice not treated or treated with doxy starting on the day of infection.

To assess if FBA is required *in vivo*, we infected mice with WT and FBA-DUC and monitored growth and survival in lungs and spleens (Figure 2). Mice infected with FBA-DUC were fed doxy-containing chow starting at the day of infection, on day 10, and on day 35 post-infection to determine whether FBA is required for growth and persistence of *Mtb*. FBA-DUC failed to replicate in lungs of mice fed with doxy starting on the day of infection and was undetectable by CFU determinations at day 10 post-infection (Figure 2A). No pathological signs of infection were observed in lungs of these mice (not shown) and no CFU were detectable in their spleens. When doxy administration was started later, CFU declined rapidly in lungs and spleens of both acutely and chronically infected mice. Decreases in CFU were accompanied by decreases in lung pathology (Figure 2B). In mice that did not receive doxy, FBA-DUC replicated and persisted similar to WT. Together these data establish that *Mtb* requires FBA activity for growth during acute and persistence during chronic mouse infections.

### FBA essentiality is carbon source-dependent

**Figure 3:**
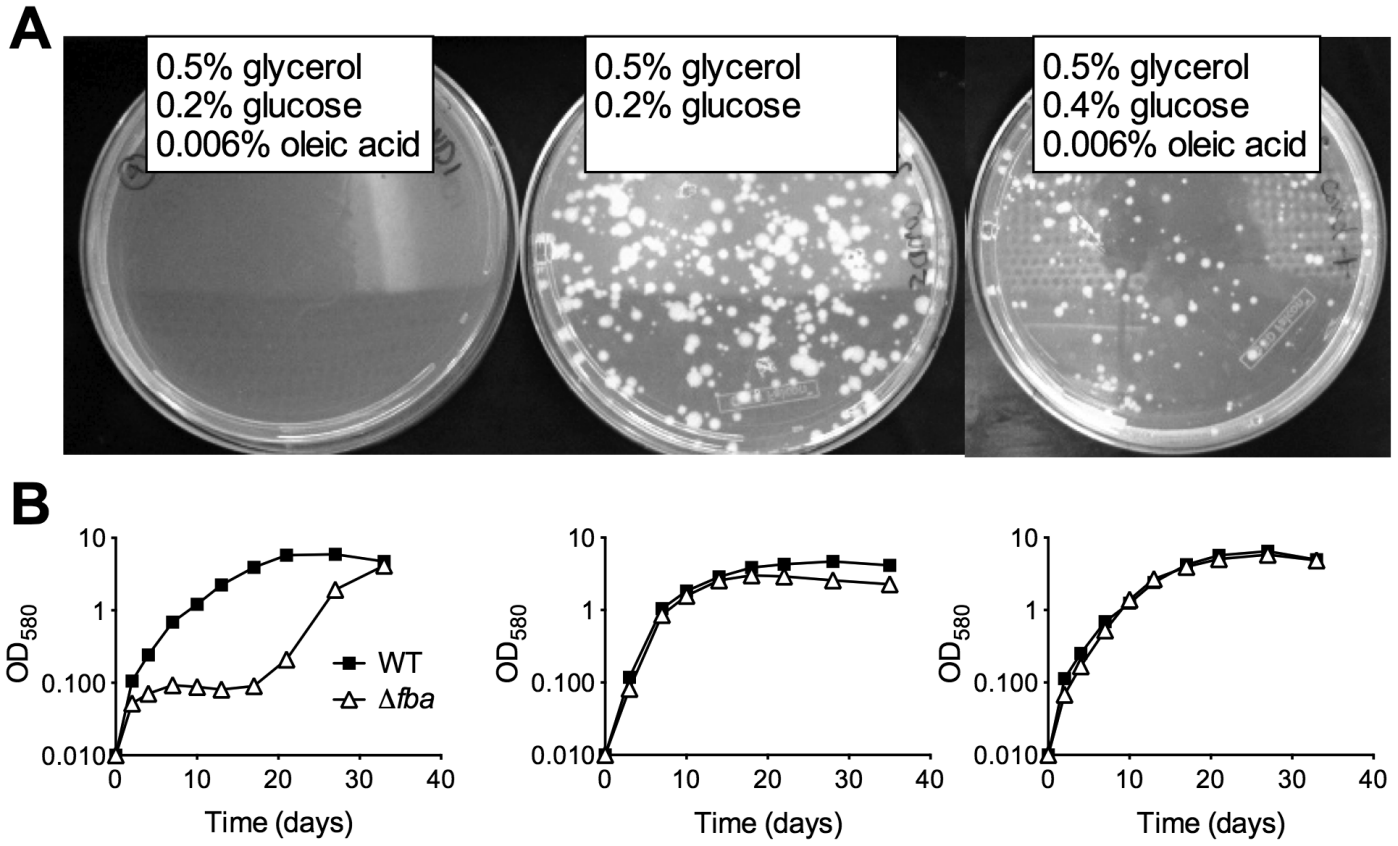
FBA essentiality is carbon source dependent. (**A**) Growth of replacement transformants of *Mtb* P_smyc_-*fba*-*fba*::hyg with a plasmid not containing *fba* and thus resulting in Δ*fba* on agar plates containing the indicated carbon sources. (**B**) Growth of Δ*fba* in 7H9 base liquid media with identical carbon sources as in the above plate conditions.

Growth of FBA-DUC with atc in dual carbon source media (Figure 1) suggested that FBA may not be essential in all conditions and that its essentiality may be carbon source-dependent. Therefore, we next attempted to delete *fba* by replacing the integrative, plasmid containing P_smyc_-*fba* with a plasmid that did not contain *fba*. Δ*fba* candidates grew readily on agar plates containing a carbon source combination - glucose and glycerol - that permitted growth of FBA-DUC with atc (Figure 3A). No Δ*fba* candidates were obtained on standard *Mtb* agar plates with glycerol and Middlebrook OADC, a supplement containing oleic acid and glucose as carbon sources. However, doubling the glucose concentration resulted in growth of colonies albeit with slower growth rate than in the absence of oleic acid (Figure 3A). Southern blot and immunoblot confirmed that the Δ*fba* candidates were true knockout strains (Figure S2, S3). As observed on agar plates, growth of Δ*fba* in liquid culture was carbon source-dependent, although Δ*fba* replicated in oleic acid-containing medium following a twenty-day lag phase (Figure 3B). This delayed growth was not from complete *fba* suppressor mutants because these bacteria were not capable of growth with a single carbon source (not shown). These experiments demonstrated that essentiality of FBA is conditional and suggested that *Mtb* lacking FBA (Δ*fba*) has specific carbon source requirements.

**Figure 4:**
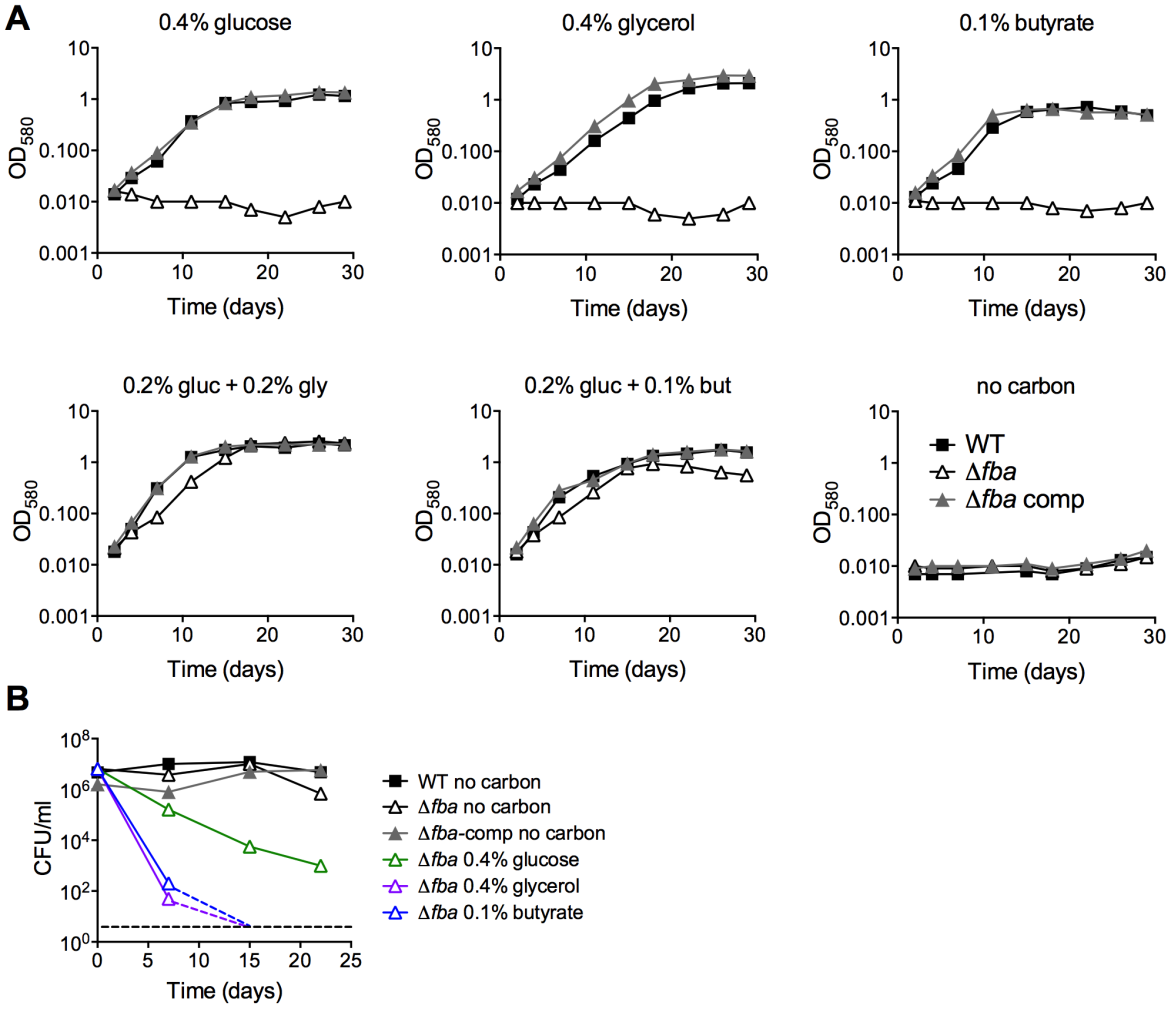
FBA is required for growth and survival in single carbon sources. (**A**) Growth measured by absorbance of WT (black squares), Δ*fba* (open triangles) and Δ*fba*-comp (grey triangles) in carbon-defined media containing glucose, glycerol and butyrate at the indicated concentrations. Bacteria were cultured in 25 cm^2^ flasks. (**B**) Survival measured by culturing for CFU on growth-permissive agar plates of the indicated *Mtb* strains in media with single carbon sources and no carbon addition at different time points post-inoculation. Stippled lines indicate that next data point was below the limit of detection.

Indeed, similar to the effects of conditional FBA-depletion, complete inactivation of FBA by deletion of *fba* caused a failure to replicate with a single carbon source but did not drastically affect growth with two carbon sources that enter central carbon metabolism above and below the FBA catalyzed step (Figure 4A). Growth of the complemented strain (Δ*fba*-comp) was similar to that of WT in all conditions tested, demonstrating that the observed growth phenotypes were caused by the lack of FBA. CFU analysis of Δ*fba* in single carbon sources revealed that Δ*fba* died rapidly, with a 5 log decrease in CFU in 7 days and no detectable CFU by day 14 in media containing glycerol, butyrate, or acetate as sole carbon sources (Figure 4B, data not shown for acetate). Death in glucose-containing medium was slower, but also substantial. In contrast, Δ*fba* survived with only a 10-fold decrease in CFU in base media without added carbon. Thus, the presence of a single carbon source resulted in killing of Δ*fba* and the kinetics of death were dependent on the specific carbon source suggesting that the mechanism leading to death of Δ*fba* in glucose may be different from that induced by glycerol or butyrate feeding below the FBA catalyzed step.

**Figure 5:**
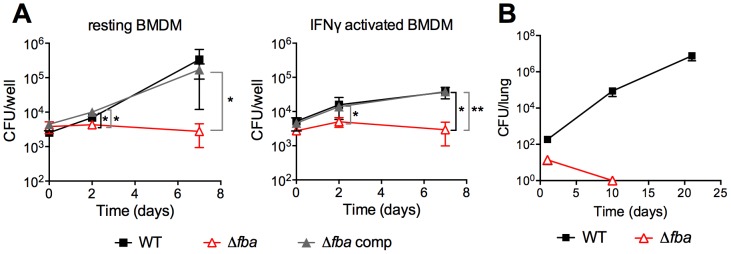
FBA is required for replication in macrophages and growth and survival in mouse lungs. (**A**) Bacterial loads in resting and IFNγ activated mouse bone marrow derived macrophages (BMDM) infected with carbon-starved WT, *Δfba* and *Δfba*-comp. Data are means +/− SD of triplicate wells. *P≤0.05; **P≤0.005 by Student's t-test (**B**) Growth and survival of WT and *Δfba* in mouse lungs. Data are means +/− SD from four mice. Limit of detection was 4 CFU and *Δfba* was not detectable on days 10 and 21.

Δ*fba* failed to replicate in resting bone marrow derived mouse macrophages (BMDM) in contrast to WT and Δ*fba*-comp (Figure 5A). Activation of macrophages with IFNγ reduced replication of WT and Δ*fba*-comp and did not affect survival of Δ*fba*. In mice, Δ*fba* did not establish an infection and was cleared from mouse lungs by day 10 post infection (Figure 5B). Thus, the metabolic environment in phagosomes of macrophages *ex vivo* and in mouse lungs did not support growth of *Mtb* in the absence of FBA and resulted in killing of Δ*fba* in mice. The lack of death of Δ*fba* in isolated macrophages suggests that the intracellular environment in macrophages *ex vivo* does not exactly mimic that faced by *Mtb* in macrophages inside the lung.

### Metabolic consequences of *fba* deletion

**Figure 6:**
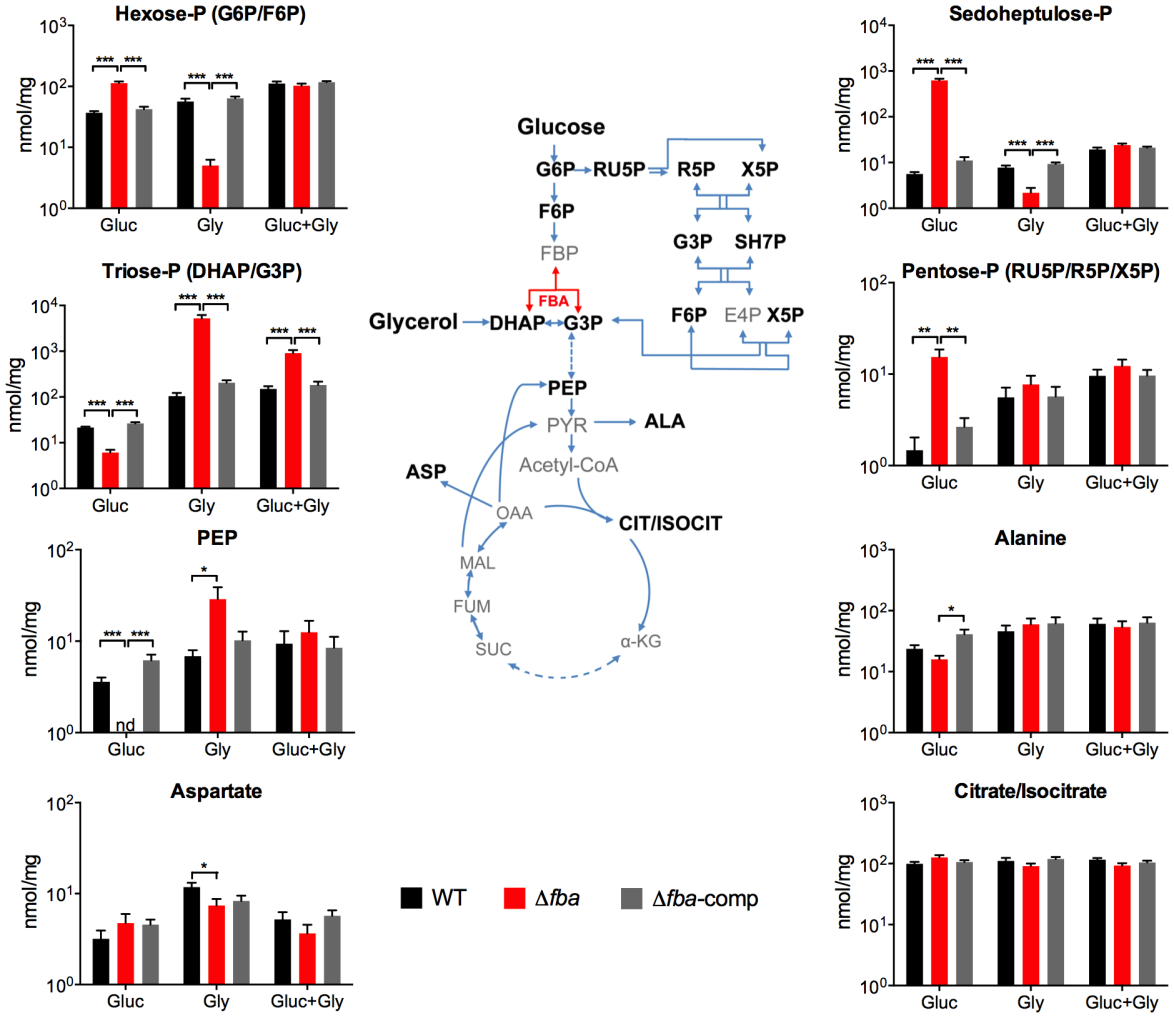
Metabolic consequences of *fba* deletion. Intrabacterial pool sizes of selected metabolites (nmol/mg protein) in the indicated *Mtb* strains after 24 hours culture on media containing either glucose (Gluc), glycerol (Gly) or a combination of both. nd = not detected. All values are means of measurements from two independent experiments, each performed with triplicate cultures ± SEM. *P≤0.05; **P≤0.005; ***P≤0.0005 by Student's t-test. ALA, alanine; α-KG, α-ketoglutarate; ASP, aspartate; CIT/ISOCIT, citrate/isocitrate; DHAP, dihydroxyacetone phosphate; E4P, erythrose 4-phosphate; FBA, fructose-1,6-bisphosphate aldolase; F6P, fructose 6-phosphate; FBP, fructose 1,6-bisphosphate; FUM, fumarate; G3P, glyceraldehyde 3-phosphate; G6P, glucose 6-phosphate; MAL, malate; OAA, oxaloacetate; PEP, phosphoenolpyruvate; PYR, pyruvate; R5P, ribose 5-phosphate; RU5P, ribulose 5-phosphate, SH7P, sedoheptulose 7-phosphate; SUC, succinate; X5P, xylulose 5-phosphate.

To better understand the impact of loss of FBA on *Mtb* metabolism we used liquid chromatography/mass spectrometry to measure metabolite levels in WT, Δ*fba*, and Δ*fba*-comp cultured on filters on top of agar plates containing 0.2% glucose, 0.2% glycerol, or a combination of 0.2% glucose and 0.2% glycerol (Figure 6). Following exposure to glucose alone, Δ*fba* accumulated high levels of hexose-phosphate (P) and depleted its triose-P and phosphoenolpyruvate (PEP) pools. These metabolic changes are consistent with a defect in glucose metabolism due to the absence of FBA activity. Pentose-P and sedoheptulose-P pools were also significantly increased in Δ*fba* suggesting either increased flux through the pentose phosphate (PP) pathway or decreased metabolism of PP pathway intermediates. In contrast, exposure to glycerol as sole carbon source resulted in depletion of hexose-P and sedoheptulose-P pools while triose-P levels were increased. The presence of both glucose and glycerol reversed most metabolic changes in Δ*fba* except for the triose-P pool, which remained elevated, but not to the same extent as with glycerol as sole carbon source. These experiments demonstrate that metabolism of single carbon sources by Δ*fba* significantly altered intracellular metabolite concentrations, which was accompanied by cell death (Figure 4). The buildup of PP pathway metabolites was consistent with increased flux into the PP pathway during culture on glucose; however, the PP pathway could not act as an efficient bypass to overcome loss of FBA.

### Only a specific balance of carbon sources can compensate for the lack of FBA

**Figure 7:**
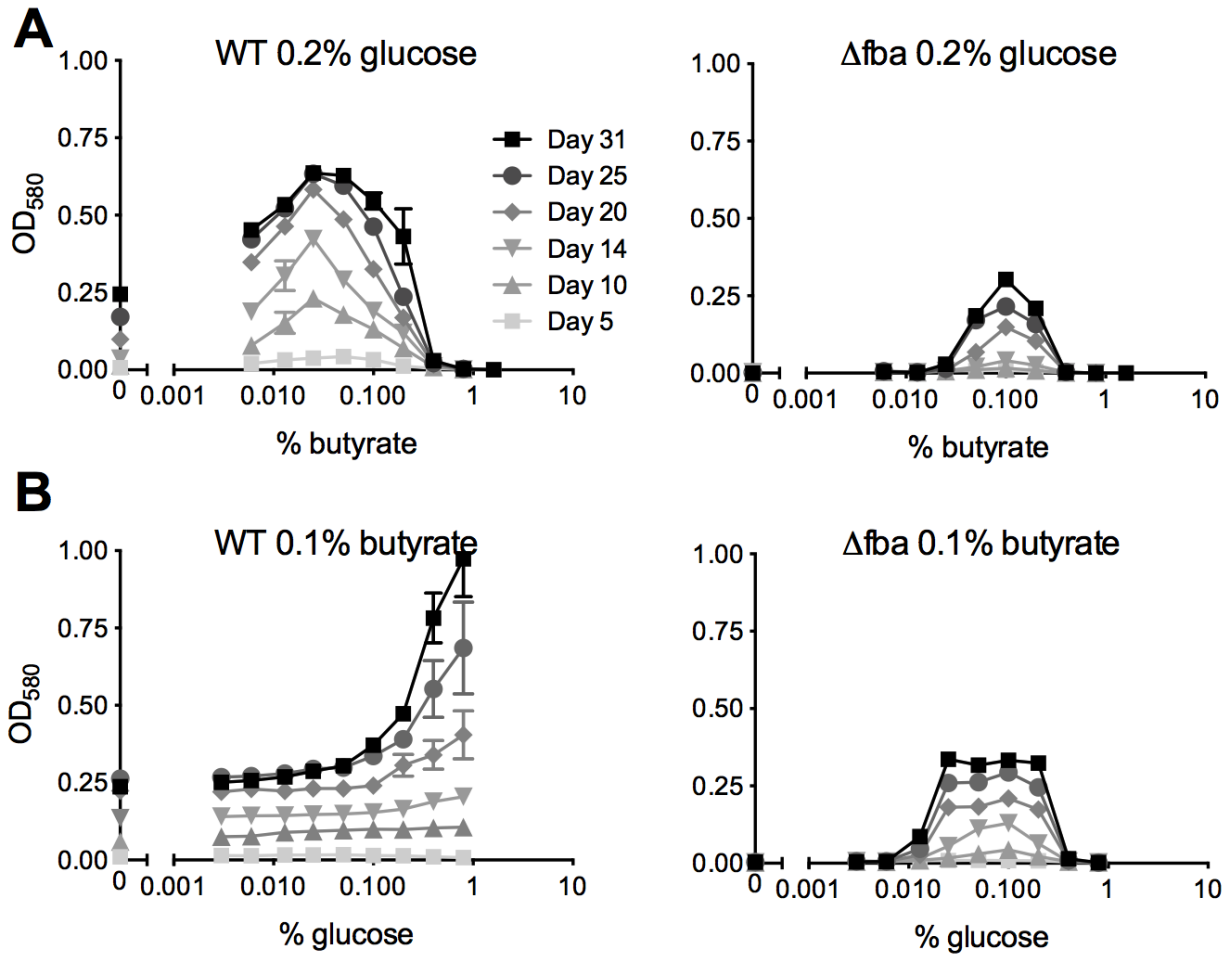
*Mtb* lacking FBA requires a balanced carbon diet for growth. Growth of WT and Δ*fba* was in media containing (**A**) 0.2% glucose with varying concentrations of butyrate and (**B**) 0.1% butyrate with varying concentrations of glucose. Bacteria were cultured in 96-well plates and absorbance was measured at the indicated time points. Data are means of triplicate cultures +/− SEM and representative of two independent experiments.

Growth of Δ*fba* required the presence of two carbon sources entering metabolism above and below the FBA catalyzed reaction and on agar plates was dependent on the amount of glucose in media containing glycerol and oleic acid (Figure 3). We therefore sought to investigate whether FBA might regulate a specific balance of glycolytic and gluconeogenic metabolism. WT *Mtb* replicated with glucose as sole carbon source and even low concentrations of butyrate enhanced its growth as previously reported [4] up to 0.4% when butyrate became toxic (Figure 7A). In contrast, when provided with 0.2% glucose, Δ*fba* required at least 0.05% butyrate for growth, which was maximal with 0.1% but did not reach that of WT suggesting that in these conditions *Mtb's* capacity to metabolize butyrate is limited by FBA deficiency.

With a fixed concentration of 0.1% butyrate, WT growth increased with increasing amounts of glucose (Figure 7B). Growth of Δ*fba*, however, plateaued with 0.025% glucose and was inhibited at concentrations above 0.2%. Inefficient glucose metabolism thus limited growth of Δ*fba* in the presence of butyrate suggesting that FBA facilitates the efficient catabolism of butyrate by driving glycolysis. Together these data imply that the efficiency of glucose metabolism determines *Mtb's* capacity to co-catabolize butyrate.

### Vulnerability of *Mtb* to partial FBA depletion depends on the carbon source

**Figure 8:**
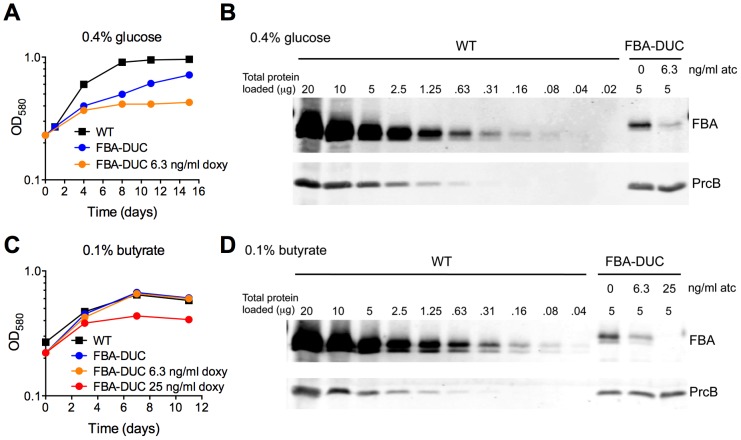
Vulnerability of *Mtb* to FBA depletion depends on the carbon source. Growth of WT and FBA-DUC without and with indicated amounts of atc in (**A**) 0.4% glucose or (**C**) 0.1% butyrate. (**B**) FBA immunoblot in protein extracts from 0.4% glucose cultures on day 15 after atc addition. (**D**) FBA immunoblot in protein extracts from 0.1% butyrate cultures on day 11 after atc addition. PrcB served as loading control.

Given the *in vivo* essentiality in acute and chronic mouse infections, our final goal was to evaluate to what degree FBA has to be depleted to result in growth inhibition and whether this is dependent on the available carbon source. FBA-DUC grew slower than WT in media with 0.4% glucose even in the absence of atc (Figure 1) and in glucose FBA-DUC was more sensitive to atc-induced growth inhibition than in butyrate-containing media (Figure S4). The reduced growth in glucose-containing, atc-free media (Figure 1,8A) was likely due to the low FBA amounts expressed in FBA-DUC, which were reduced by approximately 87% compared to WT even in the absence of atc (Figure 8B). Addition of 6.3 ng/ml atc depleted FBA by approximately 97% and abolished growth of FBA-DUC in glucose containing medium. In contrast, depletion by 87% or 97% was insufficient to reduce growth of FBA-DUC in medium with 0.1% butyrate as the sole carbon source. Addition of 25 ng/ml atc, which reduced FBA expression below the level of detection, was required to inhibit growth with butyrate. Thus, *Mtb* was more sensitive to FBA depletion when grown with glucose as sole carbon source than when metabolizing butyrate emphasizing that vulnerability to depletion of an essential protein can be dependent on the growth condition.

## Discussion

The experiments reported here enhance our understanding of *Mtb* carbon metabolism and are relevant to tuberculosis drug development. *Mtb fba* has previously been shown to be required for growth on standard media [19], [33] and FBA inhibitors are under development [22], [29], [30], but to our knowledge no FBA-specific inhibitor has been effective at inhibiting growth of live *Mtb*. Furthermore, it was unknown whether FBA is essential for growth or survival of *Mtb in vivo*. Using a genetic approach we evaluated FBA as a potential drug target by assessing its essentiality for growth and persistence during mouse infections. Depletion of FBA after establishment of chronic infection led to complete clearance of viable *Mtb* in mouse lungs and spleens, which promotes FBA as a potential therapeutic target for killing of persistent bacilli.

Unexpectedly, FBA was not essential in all conditions *in vitro*, and a conditional *fba* mutant was critical to identify conditions in which Δ*fba* could be isolated. This deletion mutant was able to replicate when provided with combinations of carbon substrates entering metabolism above and below the FBA-catalyzed reaction. Thus, it will be crucial to determine the efficacy of FBA inhibitors against live *Mtb* in carbon conditions where *Mtb* is vulnerable to FBA inactivation in contrast to standard mycobacterial liquid culture medium, which contains a combination of glucose and glycerol making FBA dispensable.

Earlier studies have highlighted *Mtb's* ability to co-catabolize multiple carbon sources *in vitro* and in macrophages [4]–[6]. This is in contrast to other bacteria such as *E. coli*, which exhibits diauxic growth in the presence of multiple carbon sources resulting from their sequential metabolism [34], [35]. Here, we provide further evidence for *Mtb's* co-catabolism and offer insight into its metabolic regulation. Growth of WT *Mtb* on glucose and on butyrate, which requires β-oxidation for conversion into acetyl-CoA mimicking the fate of long chain fatty acids, was enhanced in a dose responsive manner by addition of the respective other carbon source. Growth of Δ*fba* only occurred with two carbon substrates, feeding into either side of the FBA reaction. Additionally, deletion of FBA rendered *Mtb* extraordinarily sensitive to the relative concentrations of carbon sources in the medium. In media containing glucose and butyrate, Δ*fba* was unable to efficiently utilize glucose to enhance its growth while growth of WT scaled with glucose. Metabolism of increasing amounts of butyrate was also restricted shown by Δ*fba*'s limited growth despite the presence of 0.2% glucose. FBA thus facilitates efficient butyrate catabolism through its ability to metabolize glucose and FBA inactivation resulted in suboptimal co-catabolism of glucose and butyrate.

The mechanism of death of Δ*fba* in carbon-unbalanced media remains to be determined but may be due to buildup of FBA substrates at higher concentrations of glucose or butyrate, as well as regulatory mechanisms allowing *Mtb* to sense carbon levels and respond accordingly. Indeed, the inability of Δ*fba* to grow on standard *Mtb* plate medium containing glucose, glycerol and oleic acid was overcome by the addition of 0.2% additional glucose, which was not required for growth in nearly identical medium lacking oleic acid.

Inactivation of FBA in *E. coli* prevented growth with glucose but not with glycerol and succinate as carbon substrates [36], [37]. Accumulation of sugar phosphates including the FBA substrate fructose-1,6-bisphosphate (FBP) is thought to be the cause of this growth inhibition, which is supported by suppressor mutations that prevent FBP buildup and allow growth on glucose [38]. With our metabolite extraction protocol we did not detect FBP, however other sugar phosphates including hexose-P, sedoheptulose-P and pentose-P increased dramatically in Δ*fba* cultured on glucose, while triose-P and PEP accumulated on glycerol. The accumulation of these metabolites was not observed when both glucose and glycerol were available to Δ*fba*, except for the triose-P pools, which remained elevated but much less than in the glycerol condition. It is thus possible that the accumulation of phosphorylated metabolites is cause for the death of Δ*fba* in conditions where glycolytic and gluconeogenic carbon flow is unbalanced, such as with single carbon substrates or in mixes with an abundance of a glycolytic or gluconeogenic substrate.

How potent would an FBA inhibitor need to be to affect *Mtb* growth? We measured the vulnerability of *Mtb* to FBA depletion in single glycolytic and gluconeogenic carbon sources where FBA is essential. Although depleting FBA by 97% did not affect growth with butyrate as sole carbon source, it prevented growth with glucose and 87% inhibition was sufficient to reduce growth with glucose. These data reveal a differential susceptibility of *Mtb* to FBA depletion, depending on the available carbon source. Antibiotic targets vary widely in how much inhibition is required to stop replication or induce death and not all effective drug targets are highly vulnerable to inhibition [39]. Specific target-drug interactions can contribute to the efficacy of a compound. We cannot predict how effectively FBA must be depleted to abolish replication *in vivo*, but the WT-like phenotype of FBA-DUC without doxy suggests that 13% of WT FBA levels are sufficient for normal growth and persistence in mice. The fast killing of *Mtb* following further FBA depletion in mouse lungs and spleens suggests that it is an effective target during acute and chronic mouse infections. During infections FBA may also have an additional, metabolism-independent function in *Mtb*'s interaction with the host as it can be secreted and bind to human plasminogen [19].

The requirement of FBA for growth and persistence in mice suggests that *in vivo Mtb* either faces single carbon sources or lacks access to the growth permissive ratio of carbon sources that can compensate for the lack of FBA. This is likely due to an abundance of fatty acids and lipids which are predominant carbon sources available to *Mtb in vivo* [7]–[10]. It is unknown if FBA is essential for growth and persistence of *Mtb* in humans; experimental animal models that more closely mimic human TB pathology [40] would help address this question. Given that human granulomas contain lipid-rich foamy macrophages and build up lipids in the caseum [41], [42], it is plausible that *Mtb* requires FBA also during human infection.

## Materials and Methods

### Ethics statement

Mouse studies were performed following National Institutes of Health guidelines for housing and care of laboratory animals and performed in accordance with institutional regulations after protocol review and approval by the Institutional Animal Care and Use Committee of Weill Cornell Medical College (protocol # 0601-441A, Conditional Expression of Mycobacterial Genes).

### Strains, media and culture conditions

*M. tuberculosis* H37Rv strains were grown in Middlebrook 7H9 liquid media containing 0.5% bovine serum albumin fraction V, 0.2% glucose, 0.2% glycerol, 0.085% NaCl, and 0.05% tyloxapol without shaking in 5% CO2 at 37°C. For carbon-defined growth curves, *Mtb* was cultured in Sauton's base media modified to be carbon-limited, containing 0.05% potassium phosphate monobasic, 0.05% magnesium sulfate heptahydrate, 0.2% citric acid, 0.005% ferric ammonium citrate, 0.05% ammonium sulfate, 0.0001% zinc sulfate, and 0.05% tyloxapol at pH 7.4. For solid media, Middlebrook 7H10 media with 0.5% glycerol and 10% Middlebrook OADC supplement (final concentration of 0.5% bovine serum albumin fraction V, 0.2% glucose, 0.085% NaCl, 0.006% oleic acid, 0.0003% catalase) or self-made ADNaCl (final concentration of 0.5% bovine serum albumin fraction V, 0.2% glucose, 0.085% NaCl) was used. Carbon sources glucose, glycerol, sodium acetate and butyric acid, were added at indicated concentration (%wt/vol or %vol/vol, depending on stock). When appropriate, hygromycin B (50 µg/ml), streptomycin (10 µg/ml), kanamycin (25 µg/ml), and/or zeocin (25 µg/ml) were added. Anhydrotetracycline (atc) was added at the indicated concentrations and replenished at half the initial concentration in liquid cultures every 4–5 days for growth curves but not vulnerability assays. For survival assays, bacterial culture samples were taken from growth curve cultures at the time-points indicated and plated for CFU. For metabolomic profiling, *Mtb* was seeded at OD_580_∼1 on 0.22 µM nitrocellulose filters (1 ml per filter) and cultured on Middlebrook 7H10 agar medium containing 0.5% bovine serum albumin fraction V, 0.085% NaCl, 0.2% glucose, and 0.2% glycerol for 5 days. Filters were then transferred to similar plates with defined carbon sources: 0.2% glucose, 0.2% glycerol, or both together, each at 0.2%. *Mtb* was harvested 24 hours later by metabolic quenching in cold acetonitrile∶methanol∶H_2_O (40∶40∶20) and mechanically lysed using a bead beater as described [4], [10].

### Generation of mutant strains

*M. tuberculosis* H37Rv was transformed with a plasmid expressing *fba* under the control of a strong promoter P1 (P_smyc_ [32]) that integrates into the phage attL5 site in the *Mtb* genome. In this *fba* merodiploid strain deletion of native *fba* was achieved by allelic exchange using specialized transducing phage phAE87 as previously described [10]. After confirming removal of native *fba* by Southern blot, replacement transformations of the attL5 insets were performed to generate FBA-DUC and Δ*fba*. In FBA-DUC, *fba* contained a C-terminal DAS+4 tag and a plasmid in the phage tweety site that allowed inducible expression of *SspB* as described [31]. In Δ*fba* the attL5 site carries a kanamycin-resistant plasmid not containing *fba*. All plasmids were constructed using Gateway Cloning Technology (Invitrogen) using BP and LR recombinase reactions following the manufacturers instructions. The complemented mutant is Δ*fba* transformed with a plasmid that integrates in the attL5 site expressing *fba* from the P1 promoter.

### Metabolomics using Liquid Chromatography-Mass Spectrometry

*M. tuberculosis* metabolites were separated and detected in a Agilent Accurate Mass 6220 TOF coupled to an Agilent 1200 Liquid Chromatography system using a Cogent Diamond Hydride Type C column (Microsolve Technologies) using solvents and configuration as described [43]. Metabolites were quantified by standard curves generated with authentic chemicals spiked into homologous mycobacterial lysates. Quantified metabolite concentrations were normalized to bacterial biomass of individual samples determined by measuring residual protein content (BCA Protein Assay kit; Pierce).

### Immunoblot analysis for vulnerability assay

Protein extracts were prepared from bacterial pellets from 30 ml cultures at indicated time points in specified media. Briefly, cultures were washed with phosphate buffered saline (PBS), 0.05% Tween 80 and resuspended in 1 ml PBS, 1× protease inhibitor cocktail (Roche). Cells were lysed by bead beating three times at 4500 rpm for 30 seconds with 0.1 mm Zirconia/Silica beads. Beads and cell walls were removed through centrifugation (11000× g/10 min/4°C) and the supernatant was filtered through a 0.2 µm SpinX column (Corning). Lysate protein concentrations were determined using a DC Protein Assay Kit (Bio-Rad). For immunoblots 20-0.02 µg protein extracts were separated by SDS-PAGE, transferred to nitrocellulose membranes and probed overnight with rabbit antisera FBA [19] (1∶2000 dilution in 1∶1 PBS/Odyssey Blocking Buffer (LI-COR Biosciences), 0.1% Tween20) or PrcB (1∶18,000 dilution in 1∶1 PBS/Odyssey Blocking Buffer (LI-COR Biosciences), 0.1% Tween20). As secondary antibody IRDye 680 Donkey anti-Rabbit IgG(H+L) (LI-COR Biosciences) was used. Proteins were detected using the Odyssey Infrared Imaging System (LI-COR Biosciences).

### Mouse and macrophage infections

Female C57BL/6 mice (Jackson Laboratory) were infected by aerosol using an inhalation exposure system (Glas-Col) and early-log-phase *M. tuberculosis* cultures as single-cell suspensions in PBS to deliver 100 to 200 bacilli per mouse. Doxycycline containing food (2000 ppm, Research Diets) was given to mice starting at the indicated time-points. Serial dilutions of lungs and spleens homogenates were cultured on 7H10 plates containing ADNaCl to determine CFU at the indicated time points. The left lobe of each lung was fixed in 10% buffered formalin, further processed for histopathology and stained with hematoxilyn and eosin. We isolated and infected bone marrow-derived mouse macrophages as previously described [44].

## Supporting Information

Figure S1 **Metabolic schematic of glycolysis, gluconeogenesis and the tricarboxylic acid (TCA) cycle.** Enzymes and their encoding genes are color coded to reflect their dedicated pathways: glycolysis and pentose phosphate pathway (light blue), gluconeogenesis (red), glycolysis and gluconeogenesis (purple), TCA cycle (green), and glyoxylate shunt (orange).
(TIF)

Figure S2 **Confirmation of** ***fba*** **deletion in** ***fba*** **mutant strains.** (**A**) Genomic organization and Southern blot design with (1) native *fba* locus before and after replacement with a hygromycin resistance cassette and (2) attL5 phage integration site after integration of the plasmid containing *fba* transcribed by a strong promoter P1-*fba* (P_smyc_-*fba*). A 1 kb probe spanning Rv0364 and *fba* detects SacI-digested DNA fragments either containing the *fba* locus or *fba* elsewhere in the genome. (**B**). Southern blot showing the expected band patterns for wild type (WT) and *fba* mutants. WT, with a 4024 kb band indicative of an intact native *fba* locus, was transformed with P_smyc_-*fba*, resulting in a 8809 bp band indicative of P_smyc_-*fba* integration at the attL5 site. Then we deleted native *fba* in this merodiploid strain by replacing it with a hygromycin resistance cassette. This mutant strain expresses *fba* at the attL5 site but not in the native locus and we observed the new expected band pattern of 2787 bps. The knockout was generated by replacement transformation, in which we selected for loss of P_smyc_-*fba* (streptomycin sensitivity) and gain of a new plasmid (kanamycin resistance) not containing *fba*. Southern blot confirmed loss of the band indicative of *fba* at the attL5 site revealing only one band of 2787 bps. This knockout strain is referred to as *Δfba*.
(TIF)

Figure S3 **FBA expression in WT and mutants.** FBA (36.5 kDa) immunoblot in protein extracts from the indicated strains grown in 7H9 media with glucose and glycerol as carbon sources. In FBA-DUC the DAS+4 tag increases FBA's molecular weight by 1.6 kDa. Anhydrotetracycline (atc) was added at 500 ng/ml. FBA antiserum was applied for one hour at a 1∶3500 dilution. PrcB (30.3 kDa) served as loading control.
(TIF)

Figure S4 **Atc-induced growth inhibition in glucose and butyrate containing media.** Growth of FBA-DUC without and with indicated amounts of atc in (**A**) 0.4% glucose or (**B**) 0.1% butyrate.
(TIFF)
