# Supplementary figures and images for: Inactivation of Fructose-1,6-Bisphosphate Aldolase Prevents Optimal Co-catabolism of Glycolytic and Gluconeogenic Carbon Substrates in Mycobacterium tuberculosis
Source: PLoS Pathog. 2014 May 22;10(5):e1004144. doi: 10.1371/journal.ppat.1004144 (PMC4031216; doi:10.1371/journal.ppat.1004144)

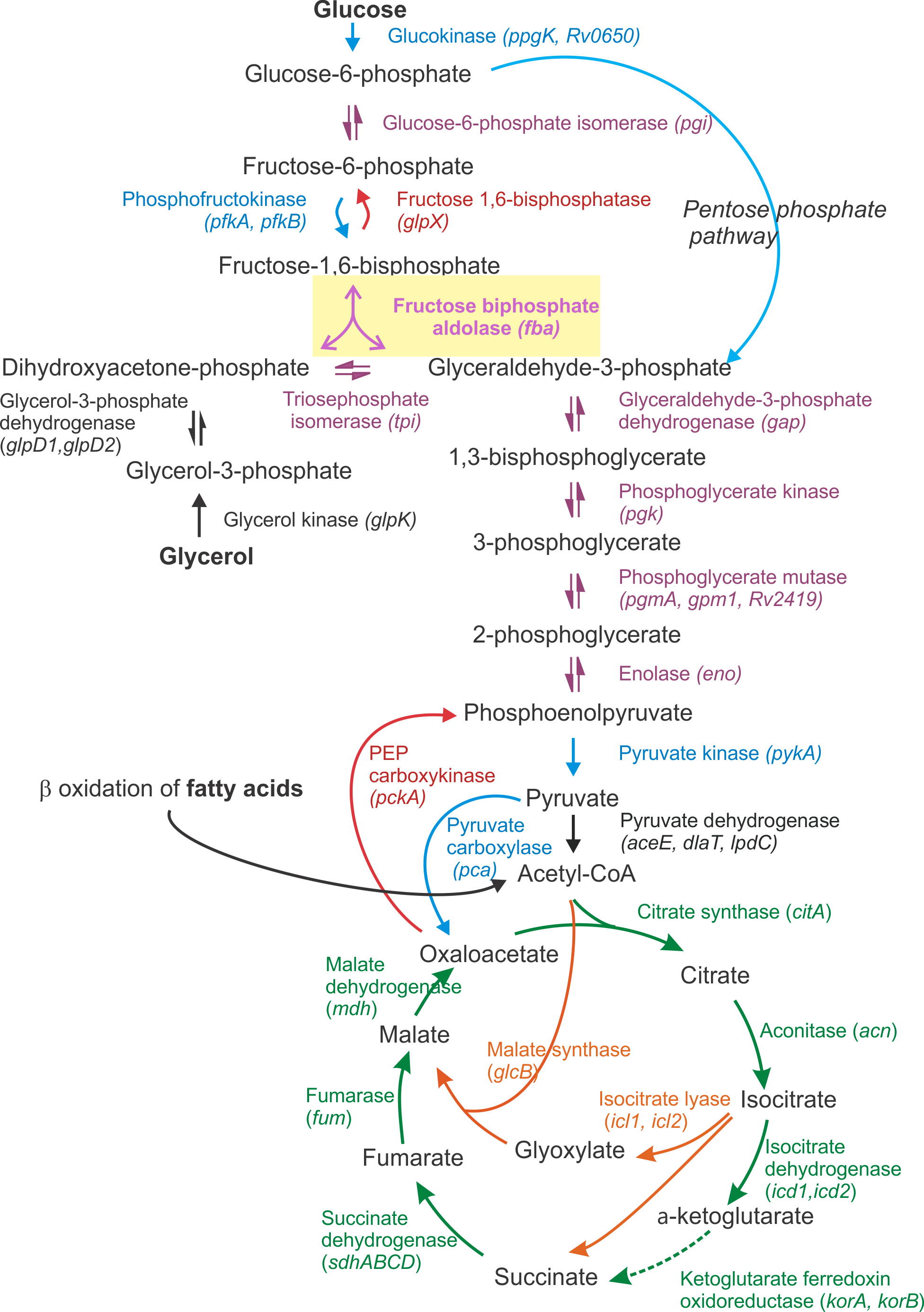

Supplement: Figure S1 — Metabolic schematic of glycolysis, gluconeogenesis and the tricarboxylic acid (TCA) cycle. Enzymes and their encoding genes are color coded to reflect their dedicated pathways: glycolysis and pentose phosphate pathway (light blue), gluconeogenesis (red), glycolysis and gluconeogenesis (purple), TCA cycle (green), and glyoxylate shunt (orange). (TIF) [file ppat.1004144.s001.tif]

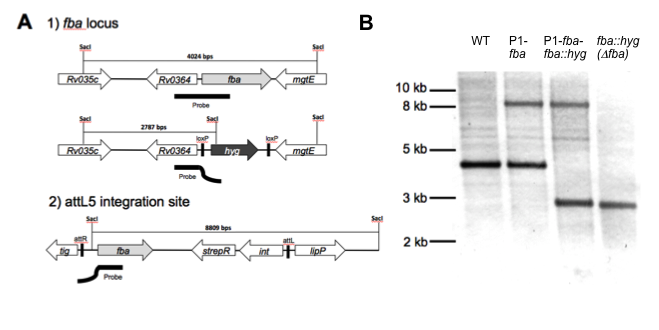

Supplement: Figure S2 — Confirmation of fba deletion in fba mutant strains. (A) Genomic organization and Southern blot design with (1) native fba locus before and after replacement with a hygromycin resistance cassette and (2) attL5 phage integration site after integration of the plasmid containing fba transcribed by a strong promoter P1-fba (Psmyc-fba). A 1 kb probe spanning Rv0364 and fba detects SacI-digested DNA fragments either containing the fba locus or fba elsewhere in the genome. (B). Southern blot showing the expected band patterns for wild type (WT) and fba mutants. WT, with a 4024 kb band indicative of an intact native fba locus, was transformed with Psmyc-fba, resulting in a 8809 bp band indicative of Psmyc-fba integration at the attL5 site. Then we deleted native fba in this merodiploid strain by replacing it with a hygromycin resistance cassette. This mutant strain expresses fba at the attL5 site but not in the native locus and we observed the new expected band pattern of 2787 bps. The knockout was generated by replacement transformation, in which we selected for loss of Psmyc-fba (streptomycin sensitivity) and gain of a new plasmid (kanamycin resistance) not containing fba. Southern blot confirmed loss of the band indicative of fba at the attL5 site revealing only one band of 2787 bps. This knockout strain is referred to as Δfba. (TIF) [file ppat.1004144.s002.tif]

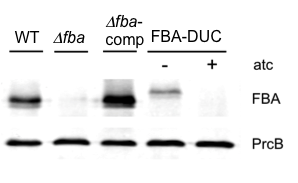

Supplement: Figure S3 — FBA expression in WT and mutants. FBA (36.5 kDa) immunoblot in protein extracts from the indicated strains grown in 7H9 media with glucose and glycerol as carbon sources. In FBA-DUC the DAS+4 tag increases FBA's molecular weight by 1.6 kDa. Anhydrotetracycline (atc) was added at 500 ng/ml. FBA antiserum was applied for one hour at a 1∶3500 dilution. PrcB (30.3 kDa) served as loading control. (TIF) [file ppat.1004144.s003.tif]

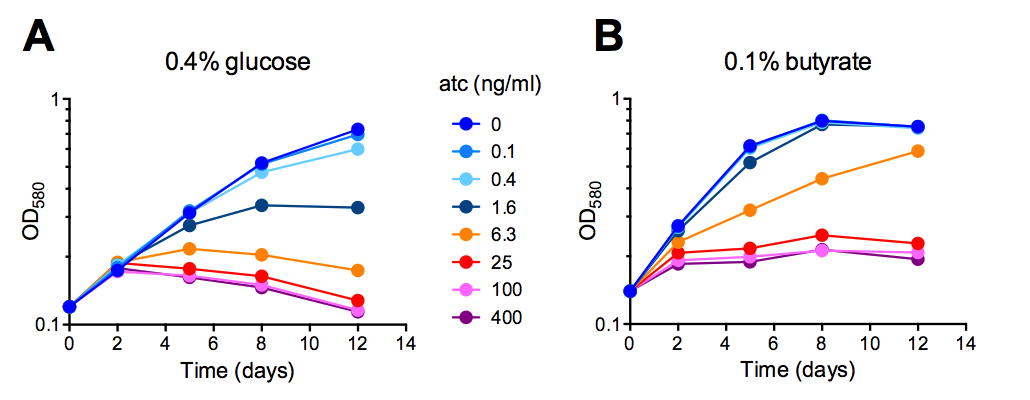

Supplement: Figure S4 — Atc-induced growth inhibition in glucose and butyrate containing media. Growth of FBA-DUC without and with indicated amounts of atc in (A) 0.4% glucose or (B) 0.1% butyrate. (TIFF) [file ppat.1004144.s004.tiff]
